# Gaze aversion in conversational settings: An investigation based on mock job interview

**DOI:** 10.16910/jemr.14.1.1

**Published:** 2021-05-19

**Authors:** Cengiz Acarturk, Bipin Indurkya, Piotr Nawrocki, Bartlomiej Sniezynski, Mateusz Jarosz, Kerem Alp Usal

**Affiliations:** Department of Cognitive Science, Middle East Technical University, Turkey; Department of Cognitive Science, Jagiellonian University, Poland; Institute of Computer Science, AGH University of Science and Technology,, Poland

**Keywords:** Gaze in conversation, gaze aversion, face contact, Human Human Interaction

## Abstract

We report the results of an empirical study on gaze aversion during dyadic human-to-human conversation in an interview setting. To address various methodological challenges in assessing gaze-to-face contact, we followed an approach where the experiment was conducted twice, each time with a different set of interviewees. In one of them the interviewer’s gaze was tracked with an eye tracker, and in the other the interviewee’s gaze was tracked. The gaze sequences obtained in both experiments were analyzed and modeled as Discrete-Time Markov Chains. The results show that the interviewer made more frequent and longer gaze contacts compared to the interviewee. Also, the interviewer made mostly diagonal gaze aversions, whereas the interviewee made sideways aversions (left or right). We discuss the relevance of this research for Human-Robot Interaction, and discuss some future research problems.

## Introduction

Non-verbal aspects of face-to-face communication have been researched intensively over the past several decades (e.g., 1, 2, 3, 4). In contrast to verbal communication, non-verbal communication covers a broad spectrum of modalities, including speech intonation, facial expressions, eye movements, and gestures.

Eye-gaze contact is a primary, non-verbal modality that has multiple functions, including initiating a conversation, regulating turn-taking, as well as signaling a topic change, among other adjustments of the roles of the interlocutors in a conversation (5, 6, 7, 8, 9, 10, see 11, for a review). The role of gaze is of particular importance in that it has its own "language" (12) as a means for establishing joint attention (13, 14) by capturing the interlocutor's attentional states through gaze directions. The partner’s eye-gaze direction results in a tendency for shifting attention, a phenomenon that has been considered reflexive and modulated by a set of social factors related to the observer (15, see 16, 17, for reviews).

Gaze aversion plays a complementary role in eye-gaze contact by regulating conversation. In terms of its specific functions in conversation, in contrast to gaze contact, gaze aversion indicates a tendency to avoid face-to-face interaction (e.g., 18, 19).

Investigations of gaze patterns in conversational settings have been conducted over the last decade by recording eye movements from a single participant or dual eye tracking, mostly in laboratory settings (20, 21, 22, 23, 24). One factor that disrupts the dual eye tracking methodology's ecological validity is the presence of head-mounted, wearable eye trackers during the conversational interaction between the interlocutors. Recent studies on the influence of the presence of glasses reveal a positive effect, in terms of attraction and attributing intelligence, on the perception of the interlocutor (see 25 for a review); however, there have been divergent findings in the past (e.g., 26, 27). Moreover, the presence of a wearable eye tracker is an artifact that may influence visual attention and the behavior of the interlocutor (28). At the moment, there seems to be no technical workaround to resolve this issue. In the present study, we tried to minimize this problem by conducting two experiments that differed in terms of which interlocutor wore the eye tracker.

An important aspect of the research on gaze aversion is the influence of eye contact on attention. Some studies (e.g., 29) have examined whether making eye contact with a person leads to a more substantial gaze-mediated orienting effect. In other studies (e.g., 30, 31), attentional boosts due to eye contact episodes (in the context of various saccadic parameters) have been investigated. Research is also being carried out on the influence of direct gaze on orienting to faces. For example, Gobel, Kim, and Richardson (32) emphasized that the gaze can signal and perceive and that this dual function needs to be considered in face perception. Mares, Smith, Johnson, and Senju (33) showed that detecting a direct gaze facilitates quick orienting to faces.

Another challenge in investigating gaze patterns in face-to-face settings is the potential influence of the interlocutors' social roles. The previous research reveals a tendency to gaze more frequently at higher-ranking interlocutors. Moreover, there is an influence of personality traits on gaze patterns in conversational settings (34, 35, 36). In the two experiments we report in this article, we aimed to control these factors by fixing the conversational setting to be a job interview. Here, an interviewer asks a set of predefined questions to an interviewee and evaluates the response of each question using a tablet, or paper and a pencil.

This approach has been employed in many past studies to investigate conversation characteristics in human-human and human-robot settings (37, 38, 39, 40). More generally, the investigation of design parameters for gaze has gained importance in Human-Robot Interaction and Social Robotics over the last decade. Recent studies show that a robots' gaze behavior significantly impacts the interaction quality, as specified in terms of a set of dimensions, such as engagement (41) and persuasiveness (42). In the context of our research, the issues related to HRI during social interaction are important, including the use of humanoid robots (which ensures reasonable experimental control) for targeting the mechanisms of joint attention (43). We believe that the findings of the present study and the implemented methodology can contribute to the domain of HRI and Social Robotics in multiple ways, including designing gaze aversion sequences and their timings during interactions (44).

In summary, the goal of the present study is to investigate the gaze patterns of the interlocutors in a specific dyadic conversation setting. In the next section, we present the study's methodology in state of the art and the challenges addressed in the present study. We then report two experiments where the participants conducted a conversation in a job interview setting. The discussion of the results and the conclusions are presented in the final section.

## Methodology

For investigating gaze patterns of interviewers and interviewees during a job-interview setting, a one-on-one interviewer-interviewee setting was established. In the first experiment (Experiment 1), we focused on the interviewers' gaze patterns while an interviewee answered a set of job interview questions. In the second experiment (Experiment 2), using the same setting as Experiment 1 but working with a different group of participants, we focused on the interviewees' gaze patterns. In both the experiments, the interviewer read the interview questions from an A4 size paper and did not say anything else. Therefore, no gaze contact was observed during the interviewer's periods of speech. The eye-tracking data was analyzed for gaze contact and aversion only for the periods in which the interviewee spoke.

The interview questions were translated from Villani (37) into the native language of the participants. We utilized a Tobii Glasses 2 wearable, binocular eye tracking device with a sampling frequency of 100 Hz and a camera with a field of view of 82 degrees in the horizontal direction and 52 degrees in the vertical direction. The reported accuracy of the device is 0.62 degrees, and it has 0.05 degrees of precision for gaze angles less than 15 degrees, and 3.05 degrees of accuracy, and 0.62 degrees of precision for gaze angles larger than 15 degrees, as published by Tobii Pro technical specifications (45). The device was used first for tracking the interviewers' gaze on the interviewees (Experiment 1) and then for tracking the interviewees' gaze on the interviewer (Experiment 2). The interview was conducted in a controlled environment with artificial lighting (160 lux illumination). The interlocutors sat about 1 to 1.5 meters from one another. The participants were asked to complete a demographic questionnaire and a TIPI test (46) for their subjective personality ratings.

The eye-tracking data were analyzed by the vendor software (Tobii Pro Lab) using the I-VT filter for automatic mapping the participants' gaze fixations with the following parameters: A window size of moving median of 3 gaze samples, 20 ms window length, 100 degrees/s threshold value, adjacent fixations not merged, fixations below 100 ms discarded.

After extracting the fixation data, four annotators annotated video recordings with gaze overlay to mark each fixation state. The fixation state was either *0,* showing gaze aversion by the interlocutor or *1* for gaze contact. For this, we mapped fixations to predefined Areas of Interest (AOIs) in the environment. The specification of an AOI is relatively straightforward in a stationary setting. However, automated analysis of eye-movement data in a dynamic setting is a well-known challenge in eye-tracking methodology (47). Solutions such as object recognition techniques for image processing have been proposed (48, 49, 50). Though the accuracy of these solutions has been increasing with the advancement of novel algorithms, each solution has its limitations. Therefore, we chose to conduct manual annotation of gaze locations to avoid errors of automated analysis. The annotators discussed and decided whether a fixation was a gaze contact or gaze aversion to provide high-quality annotations for each fixation. We identified a 100-pixel threshold for annotating the aversion fixations: a fixation was identified as an aversion fixation if it had a saccade of 100 or more pixels away from the previous fixation on an AOI.

For the annotations, the target interlocutor's entire face was labeled as "gaze contact," and the rest of the area as "gaze aversion." We should emphasize that the term "gaze contact" is a misnomer because the annotators labeled a fixation as "gaze contact" when the gaze-tracked interlocutor was gazing at the face of the other interlocutor but not precisely at the eyes. In particular, we annotated one side of the interaction. It means that the "gaze contact" in the present study included both proper "eye contact" (where people are looking at each other) and periods where the eye-tracked person is looking at the other person while they are looking away.

Given that the eye-tracking output of a specific gaze point in space is a byproduct of visualization rather than being a veridical gaze location with high precision and accuracy, and given the low functional precision of wearable eye trackers in today's technology, it is not possible to make clear discrimination between gaze contact and face contact at a distance of 1.5 meters between the interlocutors. Nevertheless, "face contact" is not a frequently used term. Therefore, we will use the term "gaze contact" or simply "contact" to mean "gaze-to-face contact" when one of the interlocutors is gazing at the other's face. Future development of high-precision wearable eye trackers may allow the measurement of gaze-to-gaze contact. However, this limitation does not apply to the content of "gaze aversion" in a similar way because the gaze-aversion region is much larger than the region occupied by the interlocutor's face. Accordingly, the use of the term "gaze aversion" is mostly correct in the eye-tracking literature.

Following the manual annotation of the fixation data, we calculated gaze durations (the sum of fixation durations) and average fixation coordinates of gaze aversions and contacts. These calculations let us know the duration of contact or aversions, frequently observed contact-aversion patterns, and the relative frequency of gaze shifts between contact and aversions. We also utilized all participants' TIPI results over five personality measures (Extroversion, Agreeableness, Conscientiousness, Emotional Stability, Openness) in our analysis.

The next step in the data analysis was evaluating gaze durations and gaze locations utilizing direct measures, such as determining the mean gaze duration on a predefined AOI and derived measures, such as computing various dispersion measures in gaze-location patterns. In particular, Hidden Markov Models (HMMs) have been employed for scanpath modeling and classification since the last decade (51). For instance, the EyePatterns software (52) conducts a pattern search to identify fixation sequences represented by strings. In this approach, a pattern is defined as a subsequence of an entire sequence of fixations. It may be expanded (with all fixations) or collapsed (repetitions of the same areas of interest are replaced by a single instance). The sequences are then analyzed to detect similar patterns using the Levenshtein distance or the Needleman-Wunsch algorithm. They can also be visualized as hierarchical trees of clusters of similar sequences.

Another gaze-sequence analysis methodology was proposed by (53), which employed differential sequence mining. The analysis was used to characterize gaze behaviors specific for individual users and particular tasks and create user-adaptive information visualizations. They also employed the EyePatterns software to find the number of occurrences of patterns. Their experimental results revealed that the frequencies of selected patterns might be used to distinguish users with low or high perceptual abilities and complicated or straightforward task types.

Hassani (54, 55) proposed a BFSPMiner pattern searching algorithm for searching patterns in a data stream without splitting it into batches. The algorithm was tested on gaze data that was recorded together with keystrokes and mouse movements. In the present study, we use the SPAM algorithm for searching gaze location sequences, which does not need splitting data into batches. In Burch (56), data mining was used for eye movement visualization by generating association rules from the data. Two types of rules were considered: *set-based rules*, in which time was not taken into account, and *sequence-based rules*, in which premises and consequences were time-ordered sequences. The sequence-based rules were hierarchically ordered in a prefix hierarchy. In the present study, we apply a methodology similar to both Hassani (54, 55) and Burch (56). However, we use sequences instead of rules. In the following sections, we report the experiments and their results.

## Experiment 1

### Participants

Fourteen interviewees (six females and eight males) and two interviewers (one male and one female) participated in this experiment. They were given a small monetary compensation (approximately 5 EUR) for their participation. All the participants (both the interviewers and the interviewees) were either undergraduate or graduate students. The interviewees' ages varied between 19 and 39 (*M* = 26.3, *SD* = 4.78). The female interviewer was 24 years old, and the male interviewer was 34 years old. One interviewer was assigned to each of the fourteen participants in a gender-balanced way so that there were four male and three female participants per interviewer.

### Procedure

A laboratory assistant welcomed the interviewees and seated them in an empty laboratory room where they filled out the consent form, the demographic data form, and a ten-item questionnaire of personality inventory (TIPI, 46). The interviewer's eye tracker calibration was completed in the interview room. Then the interviewer welcomed the interviewee and began the interview by asking a series of questions. The interviewer waited for each answer to be finished before asking the next question. The order of the interview questions was counterbalanced in blocks between the interviewees to minimize the effect of the independent variables of exposure (e.g., a full-time window of contact between the interlocutors) on the interviewers. The eye tracker recorded the interviewer’s gaze direction.

### Analysis of Fixations

The fixations were annotated with the Tobii Pro Lab analysis software. The resulting data was then analyzed by focusing on three dependent variables: The percentage of aversion durations in total duration of fixations, the percentage of gaze aversion variables in total fixation variables, and the gaze direction of the aversions. The data from three participants (three males) was removed from the analyses because their gaze contact duration and gaze contact percentage values were further than two standard derivations when z-scores were calculated.

Independent t-tests were conducted to analyze the potential influence of interviewee gender and TIPI measures on the percentage of aversion durations in all the fixation durations. This analysis revealed non-significant differences, showing that the two interviewee groups (one interviewed with the male interviewer and the other interviewed with the female interviewer) were similar in their TIPI scores (Extroversion: *t*(9) = -1.374, *p* = .203, *r* = .42, Agreeableness: *t*(9) = .064, *p* = .951, *r* = .02, Conscientiousness: *t*(9) = .029, *p* = .977, *r* = .01, Emotional Stability: *t*(9) = .484, *p* = .64, *r* = .16, Openness: *t*(9) = -.888, *p* = .398, *r* = .28). The gender of the participant did not have a significant effect on the percentage of aversion durations (*t*(9) = -0.781, *p* = .455, *r* = .25), and the percentage of gaze aversion fixations in all the fixations (*t*(9) = -0.655, *p* = .529, *r* = .21).

The majority of all the fixations (approximately 96%) were contact fixations, whereas the remaining 4% were aversion fixations. Generally, the interviewers conducted sustained face contacts (*M* = 3890 ms, *SD* = 4935 ms) when consecutive fixations on the face were counted as a single contact fixation. The average duration of single fixations during these contacts was *M* = 213 ms (*SD* = 212 ms). On the other hand, the interviewers' aversions were shorter than their contact durations (*M* = 286 ms, *SD* = 300 ms) when consecutive fixations off the face were counted as a single aversion fixation. The average duration of single fixations during aversions was *M* = 181 ms (*SD* = 114 ms). Paired samples t-test revealed that there was a significant difference between the contact and the aversion durations if consecutive fixations were accumulated, *t*(10) = 4.696, *p* = .001, *r* = .83), but not if the fixations were considered independently, *t*(10) = 1.317, *p* = .217, *r* = .39).

We compared the two interviewers in further analysis, as they had different TIPI scores: seven for the male interviewer and 3.5 for the female interviewer (out of 7). For this, we used weighted averages of fixation parameters in percentages to normalize for differences caused by the interview lengths. These weighted averages of aversion fixations were calculated as the ratios of the total number of aversion fixations relative to the total number of all fixations (henceforth, aversion fixation counts). Similarly, the weighted averages of aversion fixation durations were calculated as the ratios of the sum of aversion gaze durations relative to the sum of all gaze durations (henceforth, aversion gaze durations). A comparison between the two interviewers revealed significant differences both in their aversion fixation counts, *t*(9) = 2.287, *p* = .048, *r* = .61), and aversion gaze durations, *t*(9) = 2.708, *p* = .024, *r* = .67. The male interviewer made fewer aversions (*M* = 2.3%, *SD* = 1.7) during the interview compared to the female interviewer (*M* = 5.1%, *SD* = 2.2). The aversion gaze durations were in line with the aversion fixation counts. The female interviewer had a higher aversion duration ratio (*M* = 4.8%, *SD* = 1.7) in comparison to the male, extrovert interviewer (*M* = 2%, *SD* = 1.7). In the next section, we report a gaze sequence analysis aimed at detecting frequently observed contact-aversion patterns.

### Analysis of Gaze Sequences

We categorized the interviewers' gaze directions using labels that put consecutive fixations in the same direction as members of a single category: *C* defines a sequence of gaze *contact fixations*, whereas *Down* defines a sequence of *downward aversion fixations*. Similarly, other aversion fixations are labeled as follows: *Up* - upward, *Right* - rightward, *Left* - leftward, and *Diag* - upper or lower diagonal.

Thus, every sequence *S_j_* = (*S_1_*, *S_2_*, ..., *S_nj_*) where S_i_ ∈ Cat = {*C*, *Down*, *Up*, *Right*, *Left*, *Diag*} represents a set of consequent gaze fixations in one of the labeled directions, which occur in duration *t_i_*.

To find frequently occurring patterns, we applied the SPAM algorithm. In particular, we used the Sequential PAttern Mining (SPAM) algorithm (57) for searching gaze location sequences, which does not need splitting data into batches. The implementation of the algorithm can be found in the SPMF data mining library.^1^ The results showed that the top five frequently observed patterns in this experiment are as follows:

-
  *C Down C* (85.7%)
-
  *C Diag C* (78.6%)
-
  *C Diag C Down C* (64.3%)
-
  *C Down C Down C* (57.1%)
-
  *C Down C Diag C* (57.1%)

The numbers in the parentheses show the support value, which is defined as the percentage of gaze sequences (number of interviews) in which the pattern is found. We consider only closed patterns, which means that the sub-patterns included in more extended patterns with the same or higher support are omitted.

These findings show that the interviewers conducted downward aversions and diagonal aversions between two contact gazes. However, downward aversions are expected in the analysis, as the interviewers read questions from the paper. Although we removed question-reading segments at the beginning of each trial from our data analysis, it is still likely that the paper's presence might have resulted in a tendency towards downward fixations. Therefore, this result may be an artifact of the experimental setting. However, diagonal patterns were not observed in the interviewee aversions, as presented in the next section.

Stochastic models can be used to analyze gaze sequences. Discrete-Time Markov chain (DTMC, 58) describes a sequence of gaze directions assuming that each direction's probability depends only on the previous direction (Markov property assumption). This chain may be defined as a sequence of random variables X_1_, X_2_, X_3_, … such that

Pr(*X*_t+1_ = *x*_t+1_ | *X*_1_ = *x*_1_, *X*_2_ = *x*_2_, ..., *X*_t_ = *x*_t_) = Pr(*X*_t+1_ = *x*_t+1_ | *X*_t_=*x*_t_)

The set of outcomes *x*_i_ of the random variables is called the state space of the chain. In our case, the state space is finite and represents possible gaze directions. We also assume that the Markov chain is time-homogeneous, which means that the probabilities are independent of *t*. Therefore, they can be represented by a Markov chain transition matrix. For *n* directions, the matrix is *n x n*. The value *p*_ij_ is the probability of changing direction from *x*_i_ to *x*_j_. The values in each row of the matrix should add up to 1.

**Figure 1.**
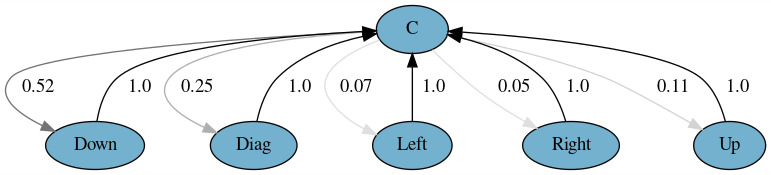
Markov model for Experiment 1 with all aversion directions distinguished.

**Figure 2.**
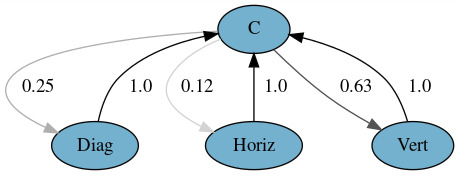
Markov model for Experiment 1 with horizontal and vertical directions aggregated.

The transition matrix for aversions in Experiment 1, with the horizontal and the vertical directions grouped, is shown in Table 1. Table 2 shows this matrix distinguishing all aversion directions. A graphical representation of these models is shown in Figures 1 and 2. These figures reveal a 52% chance of downward aversion after contact, a 25% chance of diagonal aversion, an 11% chance of upward, and a 5% of horizontal aversions. Accordingly, an aversion was always followed by a contact. That is an interesting finding since it is not usually expected to have no direct transitions between two aversions (e.g., from left to right). We will discuss this finding in the Discussion section.

**Table 1.** Chain transition matrix for Experiment 1 with horizontal and vertical directions grouped.

	Next State			
State	C	Diag	Horiz	Vert
C	0.00	0.25	0.12	0.63
Diag	1.00	0.00	0.00	0.00
Horiz	1.00	0.00	0.00	0.00
Vert	1.00	0.00	0.00	0.00

Note. The numbers show the probabilities of transitions (between 0 and 1).

**Table 2.** Chain transition matrix for Experiment 1 with all aversion directions distinguished.

	Next State					
State	C	Down	Diag	Left	Right	Up
C	0.00	0.52	0.25	0.07	0.05	0.11
Down	1.00	0.00	0.00	0.00	0.00	0.00
Diag	1.00	0.00	0.00	0.00	0.00	0.00
Left	1.00	0.00	0.00	0.00	0.00	0.00
Right	1.00	0.00	0.00	0.00	0.00	0.00
Up	1.00	0.00	0.00	0.00	0.00	0.00

Note. The numbers show the probabilities of transitions (between 0 and 1).

## Experiment 2

This experiment's setting was the same as in Experiment 1, except that we recorded the interviewees' eye movements instead of the interviewers. The male interviewer of Experiment 1 was recruited to conduct all the interviews. The stimuli (the interview questions), demographic and consent forms, and TIPI (46) questionnaire were the same as in Experiment 1.

### Participants and Procedure

Sixteen interviewees (seven females; age range 23 to 31, *M* = 26.4, *SD* = 2.85) participated in the experiment. They were offered monetary compensation (approximately 5 EUR) for their participation. All the participants were graduate or undergraduate students.

The interviewee wore the eye tracker, and its calibration was conducted before the interview in another room with similar light conditions. After the calibration, the interviewee was brought into the interview room, where the interviewer was waiting to begin the interview. The analysis procedure was also the same as for Experiment 1.

### Analysis of Fixations

The data from two male and two female participants were removed from the analyses by applying the same outlier criterion as in Experiment 1.

The majority of all the fixations (73%) were contact fixations, though to a lesser extent than the interviewer's fixations in Experiment 1 (96%). Accordingly, the remaining 27% were considered as aversion fixations. The average duration of face contacts of the interviewees (assuming consecutive fixations on the face as a single contact fixation), the average duration of single fixations (during the contacts), and the interviewees' mean aversion durations are presented in Table 3.

Paired samples t-tests revealed a significant difference between contact and aversion durations in single fixations, *t*(11) = 4.5, *p* = .001, *r* = .81, and in values where consecutive fixations of the same type are added over, *t*(11) = 3.805, *p* = .003, *r* = .75.

**Table 3.** Eye movement parameters in Experiment 1 and Experiment 2.

	Experiment 1	Experiment 2
Average contact duration (consecutive combined)	3890 (4935)	1820 (1878)
Single fixation duration in contacts	213 (212)	327 (280)
Average aversion duration (consecutive combined)	286 (300)	830 (1230)
Single fixation duration in aversions	181 (114)	242 (166)

Note. The results are in ms. The parentheses show standard deviations. For calculating average contact and aversion durations, consecutive single fixations on the same target were combined).

For assessing the influence of personality (TIPI scores) and gender on gaze parameters in Experiment 2, the data from the interviewees was divided into two groups based on their extroversion scores. Eight participants were extroverts (TIPI score in extraversion > 4) and four participants were introverts (TIPI score < 4). This distribution was similar to that obtained in Experiment 1. The TIPI measures of the interviewees did not differ between the genders (*Extroversion*: *F*(1, 11) = .457, *p* = .514; *Agreeableness*: *F*(1, 11) = .455, *p* = .515; *Conscientiousness*: *F*(1, 11) = .657, *p* = .454; *Emotional* *Stability*: *F*(1, 11) = 0, *p* = .99; *Openness*: *F*(1, 11) = 1.534, *p* = .244). The other TIPI measures were similar between the two extroversion groups (*Agreeableness*: *F*(1, 11) = 3.936, *p* = .075; *Conscientiousness*: *F*(1, 11) = 1.008, *p* = .339; *Emotional Stability*: *F*(1, 11) = .154, *p* = .703; *Openness*: *F*(1, 11) = .105, *p* = .753).

Following the same analysis procedure as Experiment 1, weighted aversion fixation counts and aversion gaze durations were compared. However, this analysis did not yield any difference between the two extroversion groups (Counts: *t*(10) = -0.763, *p* = .463, *r* = .23; Durations: *t*(10) = .902, *p* = .388, *r* = .27). A comparison of male and female interviewees did not give any statistically significant results either (Counts: *t*(10) = -1.273, *p* = .232, *r* = .37; Durations: *t*(10) = -.454, *p* = .66, *r* = .14). Pearson Correlation analysis was conducted to assess the relation between participants’ extroversion scores and gaze behavior, which did not show a significant correlation between extroversion scores and percentage of aversion counts (*r* = -.07, *p* = .84), and between extroversion scores and percentage of aversion durations (*r* = -.37, *p* = .23).

### Analysis of Gaze Sequences

The same methodology as Experiment 1 was employed for the analysis of gaze sequences in Experiment 2. The top five frequently observed patterns in Experiment 2 are listed below. The numbers in the parentheses show the support value:

-
  *C Right C* (61.5%)
-
  *C Right Left* (53.8%)
-
  *Right C Right* (53.8%)
-
  *C Right C Right* (38.5%)
-
  *C Diag Right* (38.5%)

**Figure 3.**
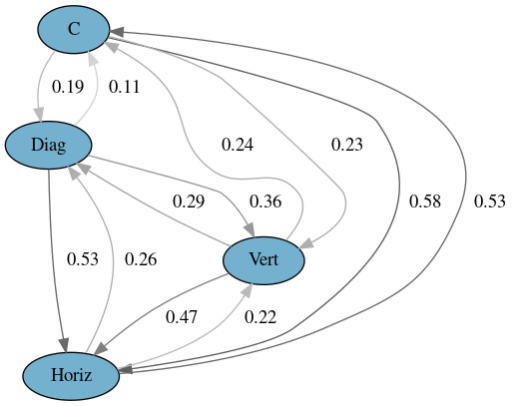
Markov model for Experiment 2 with all aversion directions distinguished.

**Figure 4.**
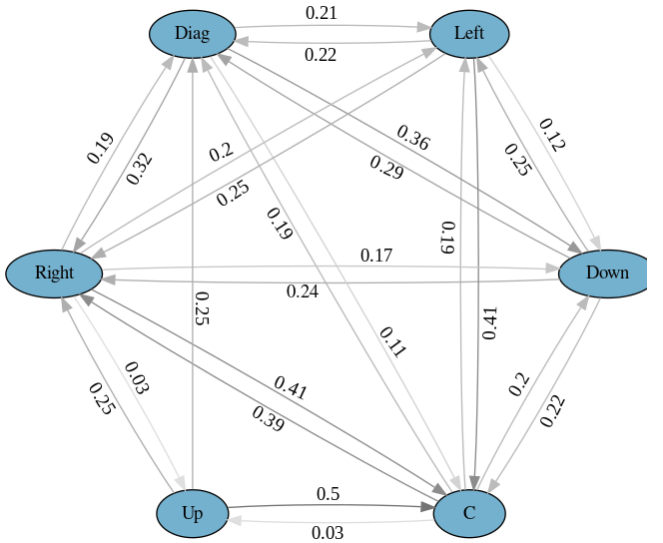
Markov model for Experiment 2 with horizontal and vertical directions aggregated.

The transition matrices for Experiment 2 are presented in Tables 4 and 5. Figures 3 and 4 show a graphical representation of the model. As expected, the interviewees did not look down so often as the interviewers because they did not have to read the questions. From the latter figure, we can see a 39% chance of right aversion after a contact, an 18-20% chance of downward, diagonal and left aversions, and only a 3% chance of upward aversion. In contrast to Experiment 1, an aversion may be followed by another aversion. After a downward aversion, left and right aversions are more probable than a contact. After a diagonal aversion, down, left, and right aversions are more probable than a contact. Another observation is that the probability distributions of the interviewee's gaze aversions (Experiment 2) are more uniformly distributed than the interviewer's gaze aversions (Experiment 1).

**Table 4.** Chain transition matrix for Experiment 2 with horizontal and vertical directions grouped.

	Next State			
State	C	Diag	Horiz	Vert
C	0.00	0.19	0.58	0.23
Diag	0.11	0.00	0.53	0.36
Horiz	0.53	0.26	0.00	0.22
Vert	0.24	0.29	0.47	0.00

Note. The numbers show the probabilities of transitions (between 0 and 1).

**Table 5.** Chain transition matrix for Experiment 2 with all aversion directions distinguished.

	Next State					
State	C	Down	Diag	Left	Right	Up
C	0.00	0.20	0.19	0.19	0.39	0.03
Down	0.22	0.00	0.29	0.25	0.24	0.00
Diag	0.11	0.36	0.00	0.21	0.32	0.00
Left	0.41	0.12	0.22	0.00	0.25	0.00
Right	0.41	0.17	0.19	0.20	0.00	0.03
Up	0.50	0.00	0.25	0.00	0.25	0.00

Note. The numbers show the probabilities of transitions (between 0 and 1).

## Discussion

When both experiments are considered, the interviewer had a lower percentage than the interviewee for both the number and the duration of aversion fixations in all the fixations during the interview. This difference was independent of the gender or extroversion score of the interviewee, which suggests that it arises from the social context of the interaction. Both the experiments were conducted in a job interview setting. In this context, the interviewer was in a socially dominant position as she or he evaluated the interviewee. It was the social dominance, but the speech duration also influenced interlocutors' gaze patterns. So, it is expected that there would be more frequent and more prolonged gaze contact fixations from the interviewer than from the interviewee.

We also found that the interviewer generally made diagonal gaze aversions, whereas the interviewees made left or right aversions with shorter durations. These findings are compatible with the previous research, which shows that gaze aversion is a robust measure in various social situations, including anxiety (59) and social phobia (60). Nevertheless, the differences in the direction of aversions between the interviewers and the interviewees are influenced by the experimental setting and the socially asymmetric situation due to the interviewer's dominance. Therefore, more studies are needed to explore these issues before generalizing these results.

A major application of this research is to design robots that show a natural gaze-aversion behavior when interacting with a human. This topic is gaining importance in Human Robot Interaction (HRI) research (see 43, for a review). The methodology of designing a robot's gaze movement based on human-to-human gaze contact studies has been followed in the past (39, 40, 61, 62). We have followed the methodology of tracking the gaze of one interlocutor at a time. We plan to evaluate our model by incorporating the interviewer's gaze contact pattern into a humanoid robot and repeat the experiment by putting the robot in the interviewer's role.

In recent years, researchers have been experimenting with deploying robots to conduct interviews (63) as they do not have unconscious biases. This approach has its problems, for a robot may show data-dependent biases. However, in this scenario, if the interviewer robot shows a natural gaze-aversion behavior based on models such as the one developed in this paper, the interviewee may feel more comfortable.

From a broader perspective, we have explored another methodology for studying gaze-contact behavior in a specific setting. As noted by Andrist (39), gaze aversion in a conversation can serve several diverse functions, such as taking turns, indicating cognitive effort, regulating intimacy. Even during the same interview, different instances of gaze aversion may play different roles. A more comprehensive model needs to take all these factors into account. Thus, it may be helpful to collect data using different methodologies, including the one proposed here.

Needless to say, our study suffers from some limitations due to the complexity of data collection in dynamic situations and our assumptions regarding data annotation and analyses. For instance, one-sided recording and data annotation constitute a limitation as the moments of "contact" in our study lumped together proper gaze contact, true face contact, and the moments where the eye-tracked interlocutor gazes the partner's face while the partner was gazing at another location. This limitation can be partially resolved by employing dual eye tracking. This solution is not perfect as dual eye tracking leads to other behavioral changes in the partners. Future research should address this problem by employing less intrusive and higher resolution eye tracking. Secondly, the results of Experiment 1 revealed a lack of direct transitions between two gaze aversions, always showing an intervening look on the interviewee's face. This finding may result from how the interviewers interacted with the interviewees, or it may be due to the low resolution of the eye tracking equipment. A higher resolution eye tracker would provide more detailed information to address this problem. Another limitation was the limited number (two) of interviewers. Though we chose one male and one female interviewer to counterbalance gender, this is insufficient to make claims about the influence of the interviewer's gender and personality on the interaction. Future research should address likely influences of such personal traits.

The annotation of fixations (as aversion fixations and contact fixations) requires operational assumptions about their definitions. In the present study, we used a 100-pixel threshold, taking into account the low resolution of the eye tracking equipment. We assumed that a fixation is an aversion fixation if it is 100 pixels away or further from the previous fixation (cf. saccadic amplitude). Fixations below the 100-pixel threshold comprised approximately a quarter of all the fixations in our data (25.5% in Experiment 1, 29.8% in Experiment 2). Further research is needed to validate this characterization of aversion fixations.

### Ethics and Conflict of Interest

The author(s) declare(s) that the contents of the article are in agreement with the ethics described in http://biblio.unibe.ch/portale/elibrary/BOP/jemr/ethics.html and that there is no conflict of interest regarding the publication of this paper.

### Acknowledgements

This research was supported in part by a grant by TUBITAK (The Scientific and Technological Research Council of Turkey) Grant No 117E021 and NCBR (The National Center for Research and Development of Poland) Grant No POLTUR2/5/2018. The authors thank Efecan Yılmaz, Gizem Özen, Elif Esmer, Gamze Eşdur, Bengisu Çağıltay and Faruk Büyüktekin for their technical support.

## Footnotes

The SPMF data mining library - http://www.philippe-fournier-viger.com/spmf/index.php.
